# Editorial Comment to Clinical characteristics of patients with inguinal hernia mesh migration into the bladder

**DOI:** 10.1002/iju5.12473

**Published:** 2022-05-15

**Authors:** Masaki Nakamura

**Affiliations:** ^1^ Department of Urology NTT Medical Center Tokyo Tokyo Japan

Akimoto *et al*. have reported two cases of hernia mesh migration to the bladder after inguinal hernia surgery.^1^ Importantly, in this manuscript, the authors have highlighted the subjective lower urinary tract symptoms and urinalysis findings caused by hernia mesh migration, as most previous cases had been reported by general surgeons and lack the urological point of view. This report recommends us to proactively consider hernia mesh migration into the bladder as a differential diagnosis for refractory lower urinary tract symptoms.

Although both mesh and nonmesh repairs are effective surgical approaches for treating hernias, mesh repair probably reduces the risk of hernia recurrence.^2^ Furthermore, when comparing laparoscopic and open surgeries, the laparoscopic technique is associated with faster recovery, lesser pain, and lower risk of hernia recurrence.^3^ Considering these results, the number of laparoscopic mesh repair surgeries is consistently increasing, when in fact, laparoscopic surgery is more prone to cause mesh migration to the bladder, as reported in this review.^1^

Notably, the required mesh size in the laparoscopic technique is larger than that in the open technique, such as the one employed in the mesh‐plug method, so the mesh can cover all the hernia gates (Husselbach's triangle, femoral ring, and inguinal ring), thus reducing the distance between the bladder and the mesh. According to this manuscript, the median period from hernia repair to diagnosis of mesh migration was 60 months,^1^ highlighting the need to continue to closely monitor morbidity trends of hernia mesh migration to the bladder.

Even if the mesh has not migrated into the bladder, adhesion of the repair site of the hernia and the bladder occasionally makes it quite difficult to expand the Retzius cavity and perform laparoscopic pelvic surgeries (e.g., robot‐assisted radical prostatectomy or robot‐assited radical cystectomy), and vice versa. Hence, it is important for urologists and general surgeons to share the possible negative effects of inguinal and pelvic surgeries and develop joint strategies.

## Conflict of interest

The author declares no conflict of interest.
